# *Candida* Species Isolation from Hospitalized Patients with COVID-19—A Retrospective Study

**DOI:** 10.3390/diagnostics12123065

**Published:** 2022-12-06

**Authors:** Petros Ioannou, Diamantis P. Kofteridis, Konstantinos Alexakis, Christos Koutserimpas, Ioanna Papakitsou, Sofia Maraki, George Samonis

**Affiliations:** 1 COVID-19 Department, University Hospital of Heraklion, 71500 Heraklion, Greece; 2 Department of Orthopaedics and Traumatology, “251” Hellenic Air Force General Hospital of Athens, 11525 Athens, Greece; 3 Department of Clinical Microbiology, University Hospital of Heraklion, 71500 Heraklion, Greece; 4 Department of Medicine, University of Crete, 71500 Heraklion, Greece; 5 First Department of Medical Oncology, “Metropolitan” Hospital, Neon Faliron, 18547 Attica, Greece

**Keywords:** *Candida*, COVID-19, SARS-CoV-2, fungal infection

## Abstract

Coronavirus disease 2019 (COVID-19), a disease characterized by severe acute respiratory syndrome coronavirus 2 (SARS-CoV-2), has so far led to hundreds of millions of infections and millions of deaths. Fungal infections are known to complicate COVID-19 patients and are associated with significant morbidity and mortality. The aim of this study was to assess the incidence of positive cultures for *Candida* spp. among patients hospitalized with COVID-19, describe their characteristics and identify factors associated with overall mortality in this patient population. Hospitalized COVID-19 patients with *Candida* spp. isolation were retrospectively assessed and their clinical, laboratory and microbiological characteristics were assessed and evaluated. In total, 69 patients with COVID-19 had a positive culture for *Candida* spp., representing a rate of 4.5% among all hospitalized COVID-19 patients. Their median age was 78 years (IQR 67–85 years) and 44.9% were male. Hospitalized patients with COVID-19 and *Candida* spp. isolation who died were older, were more likely to have a diagnosis of dementia, and had higher Charlson comorbidity index, higher *Candida* score and higher 4C score. *Candida* score was identified with a multivariate logistic regression analysis model to be independently associated with mortality. The most commonly identified *Candida* species was *C. albicans*, followed by *C. tropicalis* and *C. glabrata* and the most common source was the urine, even though in most cases the positive culture was not associated with a true infection. Thus, *Candida* score may be used in COVID-19 patients with isolation of *Candida* spp. from different body specimens for mortality risk stratification.

## 1. Introduction

Coronavirus disease 2019 (COVID-19) is the disease caused by severe acute respiratory syndrome coronavirus 2 (SARS-CoV-2) that has until now infected more than 612,000,000 people worldwide and has caused more than 6,500,000 deaths [1]. Patients with specific underlying conditions are at higher risk of hospitalization, morbidity and mortality, such as patients with diabetes, hypertension, chronic obstructive pulmonary disease, chronic kidney disease, cancer, smoking, obesity and older age, as well as patients developing medical complications such as acute kidney injury [2].

Fungal infections have been described in patients with COVID-19 and may be associated with high mortality rates in the case of invasive disease, especially in cases of mucormycosis or invasive aspergillosis [3,4,5,6]. Candidiasis is also a mycotic disease that could also, in specific circumstances, be associated with significant morbidity and mortality in patients with COVID-19, even though its prevalence among COVID-19 patients is low and is estimated at about 2.4% [7,8]. In the majority of cases, *Candida* species are isolated from urinary cultures that are of ambiguous significance [9,10]. However, invasive candidiasis, such as in the case of candidemia, may be associated with high mortality, which could be even higher for COVID-19 patients who could be at higher risk than non-COVID-19 patients for these infections [11]. More specifically, COVID-19 patients may have a higher incidence, earlier occurrence and a higher mortality rate compared to non-COVID-19 patients [11]. All these may be associated with the higher likelihood of antimicrobial use, corticosteroid and intravenous catheter use and relative immunosuppression of critically ill COVID-19 patients that could be predisposed to the development of candidiasis [12].

The aim of the present study was to assess the incidence of positive cultures for *Candida* spp. among COVID-19 patients hospitalized in a tertiary center, describe their clinical and laboratory characteristics and identify factors associated with their overall mortality, as well as the microbiological characteristics and the antifungal susceptibilities of the isolated *Candida* strains from their body specimens.

## 2. Materials and Methods

### 2.1. Study Population

In this retrospective study, participants were patients admitted to the COVID-19 Department of the University Hospital of Heraklion, Crete, Greece from the beginning of the pandemic until August 2022. Patients were included if they were 18 years old or older, were diagnosed with COVID-19 with a positive Reverse Transcription-Polymerase Chain Reaction (RT-PCR) test for severe acute respiratory syndrome coronavirus 2 (SARS-CoV-2) and had a positive culture for *Candida* spp. of body specimen during their hospital stay. Exclusion criteria were the absence of a positive RT-PCR test for SARS-CoV-2 and the transfer of patients to another ward that could not allow the completion of data collection. Data recorded and evaluated included age, gender, Infectious Diseases Society of America (IDSA) severity score, Charlson comorbidity index (CCI), 4C Score, *Candida* score, recent hospitalization, microbiological data regarding *Candida* spp. isolation, co-infection by other pathogens, admission to the Intensive Care Unit (ICU), laboratory exams, treatment administered and outcome.

The study was approved by the Ethics Committee of the University Hospital of Heraklion.

### 2.2. Microbiological Methods

For the isolation of yeasts, specimens were inoculated on Sabouraud dextrose agar supplemented with chloramphenicol (50 mg/mL) (Oxoid Limited, Hampshire, UK) and incubated at 36 °C for 72 h. The isolates were identified at the species level by matrix-assisted laser desorption/ionization time-of-flight mass spectrometry (MALDI-TOF MS) (BioMérieux, Marcy L’ Etoile, France; version 3.2). Susceptibility to antifungal agents was determined using the Vitek 2 system (BioMérieux).

### 2.3. Statistics

Categorical data were analyzed with Fisher’s exact test. Continuous variables were compared using Student’s *t*-test for normally distributed variables and the Mann–Whitney U-test for non-normally distributed variables. All tests were two-tailed and *p*-values ≤ 0.05 were considered to be significant. Data are presented as numbers (%) for categorical variables and medians [interquartile range (IQR)] or means [±standard deviation (SD)] for continuous variables. A linear regression analysis model was developed to evaluate the effect of several parameters (age, gender, IDSA severity score, CCI, 4C score, *Candida* score, recent hospitalization, admission to the ICU, laboratory exams, treatment administered, co-infections by other pathogens) with mortality. All were calculated with GraphPad Prism 6.0 (GraphPad Software, Inc., San Diego, CA, USA). A multivariate logistic regression analysis model was developed to evaluate the association of factors identified in the univariate analysis with a *p* ≤ 0.05 with mortality. Multivariate analysis was performed using SPSS version 23.0 (IBM Corp., Armonk, NY, USA).

## 3. Results

In total, 1536 patients with COVID-19 were hospitalized in the COVID-19 Department from the beginning of the pandemic until the end of August 2022. Of these, 56.1% were male and the median age was 68 years (IQR 54–79 years), while the median duration of stay was 6 days (IQR 4–10 days) and overall mortality was 7.9%. In 69 hospitalized COVID-19 patients (4.5% among all such patients), *Candida* spp. were isolated from at least one site. The median age of these 69 patients was 78 years (IQR 67–85 years) and 44.9% were male. The median duration of hospitalization was 22 days (IQR 12–30 days) and overall mortality was 34.8%. Compared to the rest of the COVID-19 patients, those with positive *Candida* spp. cultures were older (*p* < 0.0001) and had a higher duration of hospitalization (*p* < 0.0001), while overall mortality was higher (*p* < 0.0001). Table 1 and Table 2 show the characteristics of patients hospitalized with COVID-19 who had a positive culture for *Candida* spp. The tables also show the characteristics of those who survived and those who died. More specifically, a statistical analysis among hospitalized COVID-19 patients with *Candida* spp. isolation who survived with those who died showed that patients who died were older, were more likely to have a diagnosis of dementia, and had higher Charlson comorbidity index, higher *Candida* score and higher 4C score.

In terms of microbiology, 78 strains of *Candida* were isolated by 78 cultures from 69 patients hospitalized with COVID-19. The most commonly isolated species were *C. albicans*, *C. tropicalis* and *C. glabrata*. Table 3 shows the microbiology and antifungal resistance of isolated *Candida* species, while Table 4 shows the distribution of *Candida* strains according to the type of clinical specimen that yielded the strain. The source of positive culture was the urine in 60 patients (87%), the urine and blood in 3 (4.3%), the blood in 3 (4.3%) and bronchial secretions in 3 (4.3%). Of the 60 patients with positive urine cultures, only in 3 had there been a positive repeat urine culture and were considered to truly have urinary tract infection by *Candida* spp. Thus, the rate of patients that were considered to have candidiasis among all COVID-19 patients was 0.8%.

A univariate regression analysis revealed that in hospitalized COVID-19 patients with isolation of *Candida* spp. age, Charlson comorbidity index, 4C score and *Candida* score were associated with increased overall mortality. However, in a multivariate logistic regression analysis, only *Candida* score was found to be independently associated with increased overall mortality. The results of the logistic regression analysis are shown in Table 5.

## 4. Discussion

The present study investigating hospitalized COVID-19 patients has shown that *Candida* spp. was isolated at a rate of 4.5%. Most isolates were found in urinary samples and did not represent true infection. *C. albicans* was the most commonly identified species. Patients with positive *Candida* spp. cultures were older, had a higher duration of hospital stay and also had higher overall mortality. Among these patients, *Candida* score was independently associated with higher mortality.

Fungal infections in COVID-19 patients are well described and are known to be associated with excess morbidity and mortality, especially in the case of invasive disease, such as mucormycosis and invasive aspergillosis [3,4,5,6]. Moreover, COVID-19 has been considered to increase susceptibility to fungal infection in general and more specifically, to candidiasis [13,14,15]. A recent systematic review estimated the prevalence of fungal infections in COVID-19 patients at 3.7% for aspergillosis, 2.4% for candidiasis and 0.4% for other fungal infections [8]. In the present study, the rate of *Candida* spp. isolation was 4.5%. However, the majority of those cultures were not indicative of a true infection, since most of them were associated with only a single positive urine culture of ambiguous clinical significance [9,10]. After excluding patients with colonization, only 0.8% of patients were considered to truly have candidiasis.

COVID-19 may increase the likelihood of candidiasis through multiple mechanisms such as the need for hospitalization, admission to the critical care unit, intravenous catheters, corticosteroid and antimicrobial use, while, from a pathophysiological point of view, the reduction of lymphocytes seen in patients with COVID-19 possibly makes them more susceptible to this infection [12,16]. Furthermore, hyper-inflammation, which is noted in patients with COVID-19 and is characterized by high levels of pro-inflammatory cytokines in the circulation, such as interleukin-6 (IL-6), IL-1β and tumor necrosis factor, may lead to the establishment of a highly permissive environment for the development of fungal infections by facilitating damage to the host, as the exacerbated production of these cytokines may disrupt homeostasis of the immune system and lead to its pathogenic activation, leading to tissue damage and fungal invasion [15].

COVID-19 patients with isolation of *Candida* spp. were older and had a higher duration of hospitalization and higher mortality compared to the rest of the COVID-19 patients. Indeed, candidiasis may be more frequent in critically ill non-COVID-19 patients, who are commonly older, have longer hospital stays and have higher mortality compared to patients who are not critically ill [7,17,18,19]. In the present study, among hospitalized COVID-19 patients with *Candida* spp. isolation, patients who died were more likely to be old, and have higher Charlson comorbidity index, 4C and *Candida* score. A multivariate logistic regression analysis identified *Candida* score to be independently associated with mortality. *Candida* score is a score described recently and seems to have a role in identifying medically ill patients with a possibility of candidiasis [20,21]. Moreover, in a recent study, *Candida* score was identified as a predictor of mortality in patients with candidiasis, a finding in line with the results of the present study [22]. Interestingly, dementia was also identified in the univariate logistic regression analysis to be associated with overall mortality in patients with COVID-19 and *Candida* infection. However, this was not confirmed with the multivariate logistic regression analysis. There is literature, however, suggesting that dementia may be a predictor for mortality among patients with COVID-19 infection [23,24].

Regarding microbiology, the most commonly isolated species overall was *C. albicans* and it was the predominant pathogen in all different clinical samples, even though the number of isolates identified in blood and bronchial secretions was inadequate to allow the drawing of safe conclusions. This is in contrast to the findings of a study conducted in our hospital where the most commonly identified species were non-*albicans Candida* species in a general patient population [25]. This difference may be associated with the higher rate of urine cultures that were isolated in the present study, while, in the previous study, conducted in the same hospital examining 10-year data, urine cultures were excluded. No significant differences were noted in terms of antifungal resistance in the isolated strains in these two studies. Conversely, the occurrence of candidiasis in COVID-19 patients has been found to be associated with differences in antifungal resistance in recent studies [26,27]. It is of note that antifungal resistance, in particular, and antimicrobial resistance, in general, should be approached in the context of an understanding of the local patterns of resistance and antimicrobial prescribing, since these are interconnected and may lead to differences in resistance in different geographical regions [28]. 

Moreover, in other studies including COVID-19 patients, *C. albicans* was again the most commonly isolated pathogen [29]. However, the emerging pathogen *C. auris* has been also identified and was also found to predominate in specific geographical areas [7]. In the present study, *C. auris* was identified in only one culture. It is possible that the infection control measures that apply in the era of the COVID-19 pandemic could have altered the spread of this emerging fungus [30,31].

This study has some limitations, such as the fact that the patient population derives from only one hospital; thus, the generalization of the results may be limited. Furthermore, some patients who were transferred to other departments before discharge may have been missed during the evaluation.

## 5. Conclusions

In the present study, *Candida* spp. was isolated in 4.5% of hospitalized COVID-19 patients. The most commonly identified species was *C. albicans,* followed by *C. tropicalis* and *C. glabrata*. Most isolates were found in urinary samples and did not represent true infection. Patients with positive *Candida* spp. cultures were older, had a higher duration of stay in the hospital and also had higher overall mortality. Among these patients, *Candida* score was independently associated with higher mortality. Thus, *Candida* score may be useful for mortality risk stratification of COVID-19 patients with positive *Candida* spp. cultures from their body specimens.

## Figures and Tables

**Table 1:** Clinical characteristics of hospitalized patients with COVID-19 and isolation of *Candida* spp.

Characteristic	All Patients (n = 69)	Survived (n = 45)	Died (n = 24)	*p*
Age in years, median (IQR)	78 (67–85)	76 (60–81.5)	84.5 (77.3–88)	0.0002
Male gender, n (%)	31 (44.9)	22 (48.9)	9 (37.5)	0.4494
Recent hospitalization, n (%)	28 (40.6)	19 (42.2)	9 (37.5)	0.7994
Coronary artery disease, n (%)	8 (12.3)	3 (6.8)	5 (23.8)	0.0997
Heart failure, n (%)	14 (21.2)	7 (15.9)	7 (31.8)	0.2010
Dementia, n (%)	17 (25.8)	7 (15.9)	10 (45.5)	0.0161
Diabetes mellitus, n (%)	17 (25.8)	10 (22.7)	7 (31.8)	0.5518
Charlson score, median (IQR)	5 (4–8)	5 (3–8)	6 (5–8.8)	0.0384
Duration of hospitalization in days, median (IQR)	22 (12–30)	21 (11–30)	23 (12.5–34.5)	0.4887
*Candida* score, median (IQR)	0 (0–2.038)	0 (0–2.038)	2.038 (2.038–2.038)	<0.0001
4C score, median (IQR)	13 (11–15)	12 (10–14)	15 (13–17)	0.0006
Antimicrobial use at the time of positive culture, n (%)	58 (85.3)	36 (81.8)	22 (91.7)	0.1206
Urine catheter at the time of positive culture, n (%)	61 (89.7)	38 (86.4)	23 (95.8)	0.4074
Site of *Candida* isolation				0.3161
Urine, n (%)	60 (87)	37 (82.2)	23 (95.8)	
Blood and urine, n (%)	3 (4.3)	3 (6.7)	0 (0.0)	
Blood, n (%)	3 (4.3)	2 (4.4)	1 (4.2)	
Bronchial secretions, n (%)	3 (4.3)	3 (6.7)	0 (0)	
Culture-positive co-infection, n (%)	32 (46.4)	19 (42.2)	13 (54.2)	0.4483

IQR: interquartile range.

**Table 2:** Laboratory characteristics of hospitalized patients with COVID-19 and isolation of *Candida* spp.

Characteristic	All Patients (n = 69)	Survived (n = 45)	Died (n = 24)	*p*
WBC while culture positive in K/μL, median (IQR)	7.6 (4.7–11.3)	7.7 (4.7–10.7)	7.2 (4.7–11.7)	0.7904
Neutrophils while culture positive in K/μL, median (IQR)	6.8 (4.4–10.9)	6.6 (4.4–9)	9.1 (4.8–15.8)	0.0642
Lymphocytes while culture positive in /μL, median (IQR)	800 (500–1450)	800 (500–1650)	600 (325–1075)	0.0753
Hemoglobin while culture positive in g/dL, median (IQR)	10.4 (9.4–11.9)	10.7 (9.5–11.8)	10.2 (9.3–12.5)	0.9975
Platelets while culture positive in K/μL, median (IQR)	217,000 (127,500–309,000)	240,000 (136,000–321,500)	180,000 (106,300–307,000)	0.2549
LDH while culture positive in U/L, median (IQR)	344 (260.3–414.8)	340.5 (251.3–418)	346 (267.8–402)	0.8460
CRP while culture positive in ng/mL, median (IQR)	7.6 (3.2–11.9)	8.5 (4.7–14)	7.4 (3.2–11.9)	0.5599
Procalcitonin while culture positive in ng/mL, median (IQR)	0.18 (0.1–0.64)	0.19 (0.1–0.8)	0.15 (0.1–0.27)	0.6928
Interleukin-6 while culture positive in pg/mL, median (IQR)	42.5 (22.2–129.1)	44.3 (31.6–138.2)	22.2 (18.3–771.4)	0.6436
Albumin while culture positive in g/dL, median (IQR)	2.8 (2.4–3.2)	2.9 (2.5–3.2)	2.8 (2.3–3.2)	0.3610

CRP: c-reactive protein; IQR: interquartile range; LDH: lactate dehydrogenase; WBC: white blood cell count.

**Table 3:** Microbiology and resistance patterns of *Candida* strains isolated from hospitalized COVID-19 patients.

Strain	Amphotericin B, n (%)	Caspofungin, n (%)	Fluconazole, n (%)	Flucytosine, n (%)	Micafungin, n (%)	Voriconazole, n (%)
*Candida albicans* (n = 41)	S 40 (97.6) I 1 (2.4) R 0 (0)	S 39 (95.1) R 2 (4.9)	S 36 (87.8) SDD 2 (4.9) R 3 (7.3)	S 38 (92.7) I 1 (2.4) R 2 (4.9)	S 39 (95.1) R 2 (4.9)	S 40 (97.6) R 1 (2.4)
*Candida tropicalis* (n = 12)	S 12 (100) R 0 (0)	S 12 (100) R 0 (0)	S 11 (91.7) SDD 1 (8.3) R 0 (0)	S 12 (100) R 0 (0)	S 12 (100) R 0 (0)	S 12 (100) R 0 (0)
*Candida glabrata* (n = 9)	S 9 (100) R 0 (0)	S 3 (33.3) I 3 (33.3) R 3 (33.3)	S 7 (77.8) R 2 (22.2)	S 9 (100) R 0 (0)	S 9 (100) R 0 (0)	S 9 (100) R 0 (0)
*Candida parapsilosis* (n = 6)	S 6 (100) R 0 (0)	S 6 (100) R 0 (0)	S 6 (100) R 0 (0)	S 6 (100) R 0 (0)	S 6 (100) R 0 (0)	S 6 (100) R 0 (0)
*Candida krusei* (n = 4)	S 4 (100) R 0 (0)	S 1 (25) I 3 (75) R 0 (0)	S 0 (0) R 4 (100)	S 0 (0) R 4 (100)	S 4 (100) R 0 (0)	S 4 (100) R 0 (0)
*Candida lusitaniae* (n = 3)	S 3 (100) R 0 (0)	S 3 (100) R 0 (0)	S 3 (100) R 0 (0)	S 3 (100) R 0 (0)	S 3 (100) R 0 (0)	S 3 (100) R 0 (0)
*Candida guilliermondii* (n = 1)	S 1 (100) R 0 (0)	S 0 (0) R 1 (100)	S 1 (100) R 0 (0)	S 1 (100) R 0 (0)	S 1 (100) R 0 (0)	S 1 (100) R 0 (0)
*Candida kefyr* (n = 1)	S 1 (100) R 0 (0)	S 0 (0) R 1 (100)	S 1 (100) R 0 (0)	S 1 (100) R 0 (0)	S 1 (100) R 0 (0)	S 1 (100) R 0 (0)
*Candida auris* (n = 1)	S 1 (100) R 0 (0)	S 0 (0) R 1 (100)	S 0 (0) R 1 (100)	S 1 (100) R 0 (0)	S 1 (100) R 0 (0)	S 0 (0) R 1 (100)

I: Intermediate; R: resistant; S: susceptible; SDD: susceptible dose response.

**Table 4:** *Candida* species distribution in regards to the clinical specimen.

Strain	Urine (n = 69)	Blood (n = 6)	Bronchial Secretions (n = 3)
*Candida albicans*, n (%)	34 (49.3)	4 (66.7)	3 (100)
*Candida tropicalis*, n (%)	12 (17.4)	0 (0)	0 (0)
*Candida glabrata*, n (%)	8 (11.6)	1 (16.7)	0 (0)
*Candida parapsilosis*, n (%)	5 (7.2)	1 (16.7)	0 (0)
*Candida krusei*, n (%)	4 (5.8)	0 (0)	0 (0)
*Candida lusitaniae* (n = 3)	3 (4.3)	0 (0)	0 (0)
*Candida guilliermondii* (n = 1)	1 (1.4)	0 (0)	0 (0)
*Candida kefyr* (n = 1)	1 (1.4)	0 (0)	0 (0)
*Candida auris* (n = 1)	1 (1.4)	0 (0)	0 (0)

**Table 5:** Logistic regression of mortality among hospitalized COVID-19 patients with *Candida* isolation.

Characteristic	Univariate Analysis *p*	Multivariate Analysis *p*	OR (95% CI)
Age per year	0.001	0.267	1.057 (0.958–1.167)
Charlson score per unit	0.0246	0.701	1.072 (0.75–1.534)
4C Score per unit	0.0007	0.229	1.231 (0.877–1.728)
*Candida* score per unit	<0.0001	0.004	6.05 (1.775–20.625)

CI: confidence interval; OR: odds ratio.

## Data Availability

The data presented in this study are available on request from the corresponding author.
